# Genome-wide association study and genomic prediction for resistance to brown planthopper in rice

**DOI:** 10.3389/fpls.2024.1373081

**Published:** 2024-03-21

**Authors:** Cong Zhou, Weihua Jiang, Jianping Guo, Lili Zhu, Lijiang Liu, Shengyi Liu, Rongzhi Chen, Bo Du, Jin Huang

**Affiliations:** ^1^Oil Crops Research Institute of the Chinese Academy of Agricultural Sciences/The Key Laboratory of Biology and Genetic Improvement of Oil Crops, The Ministry of Agriculture and Rural Affairs, Wuhan, China; ^2^State Key Laboratory of Hybrid Rice, College of Life Sciences, Wuhan University, Wuhan, China; ^3^Cash Crops Research Institute, Hubei Academy of Agricultural Sciences, Wuhan, China

**Keywords:** rice, brown planthopper, GWAS, candidate genes, genomic prediction

## Abstract

The brown planthopper (BPH) is the most destructive insect pest that threatens rice production globally. Developing rice varieties incorporating BPH-resistant genes has proven to be an effective control measure against BPH. In this study, we assessed the resistance of a core collection consisting of 502 rice germplasms by evaluating resistance scores, weight gain rates and honeydew excretions. A total of 117 rice varieties (23.31%) exhibited resistance to BPH. Genome-wide association studies (GWAS) were performed on both the entire panel of 502 rice varieties and its subspecies, and 6 loci were significantly associated with resistance scores (P value < 1.0e^-8^). Within these loci, we identified eight candidate genes encoding receptor-like protein kinase (RLK), nucleotide-binding and leucine-rich repeat (NB-LRR), or LRR proteins. Two loci had not been detected in previous study and were entirely novel. Furthermore, we evaluated the predictive ability of genomic selection for resistance to BPH. The results revealed that the highest prediction accuracy for BPH resistance reached 0.633. As expected, the prediction accuracy increased progressively with an increasing number of SNPs, and a total of 6.7K SNPs displayed comparable accuracy to 268K SNPs. Among various statistical models tested, the random forest model exhibited superior predictive accuracy. Moreover, increasing the size of training population improved prediction accuracy; however, there was no significant difference in prediction accuracy between a training population size of 737 and 1179. Additionally, when there existed close genetic relatedness between the training and validation populations, higher prediction accuracies were observed compared to scenarios when they were genetically distant. These findings provide valuable resistance candidate genes and germplasm resources and are crucial for the application of genomic selection for breeding durable BPH-resistant rice varieties.

## Introduction

The cultivated rice (*Oryza sativa* L.) is a major staple crop and feeds over half of the global population. Rice is highly diverse, encompassing two major subspecies, *indica* and *japonica*, as well as *circum*-aus and *circum*-basmati (Wang et al., 2018a). The brown planthopper (*Nilaparvata lugens* Stål, BPH) is one of the most devastating insect pests of rice, and widely distributed in South Asia, Southeast Asia, East Asia, Northern Australia and the South Pacific Islands (Jing et al., 2017). BPH, a phloem-feeding insect, causes extensive wilting, yellowing and lethal drying of rice by sucking susceptible rice phloem sap. BPH may also indirectly damage rice plants by transmitting viruses such as grassy stunt and ragged stunt (Zheng et al., 2021). Presently, insecticides are widely used to manage pest infestations. However, this approach has damaged natural enemies and led to insecticide resistance in the insects. The most economical and effective strategy to control BPH pest is to exploit resistance genes and cultivate BPH-resistant rice varieties. Rice varieties carrying resistance genes *Bph1* or *bph2* have been implemented extensively in Southeast Asia (Jairin et al., 2007). Nevertheless, they have lost their resistance to BPH, and new BPH biotypes developed (Kobayashi, 2016). Developing rice varieties with durable resistance to BPH remains a major challenge.

The resistance mechanism of rice to BPH can be divided into antibiosis, tolerance, and antixenosis from the physiological perspective (Qiu et al., 2011). To date, over 49 BPH-resistant genes/quantitative trait loci (QTLs) have been identified and 17 BPH-resistant genes have been isolated in rice (Shi et al., 2023). Among the mapped genes, *Bph14*, *Bph25*, *Bph30* and *Bph32* were reported to confer resistance via antibiosis (Du et al., 2009; Myint et al., 2012; Ren et al., 2016; Wang et al., 2018b); *bph7*, *Bph28* and *Bph37* were considered to confer tolerance to BPH (Qiu et al., 2014; Wu et al., 2014; Yang et al., 2019); *Bph6*, *Bph9*, *Bph18*, *Bph27*, *Bph27(t)*, *Bph33* and *Bph36* confer resistance through a combination of antibiosis and antixenosis (He et al., 2013; Huang et al., 2013; Ji et al., 2016; Zhao et al., 2016; Guo et al., 2018; Hu et al., 2018; Li et al., 2019); *bph39(t)*, *bph40(t)* through a combination of antibiosis and tolerance (Akanksha et al., 2019); and *Bph31* through a combination of antibiosis, antixenosis and tolerance (Prahalada et al., 2017). The seedling bulk test is extensively used to evaluate resistance scores of rice varieties to BPH. The resistance score obtained from the seedling bulk test was a comprehensive indicator of antibiosis, tolerance and antixenosis (Qiu et al., 2011). Almost all of the BPH resistance genes were detected by the seedling bulk test.

Bi-parental populations had a narrow genetic background, restricting the detection of abundant genes. Genome-wide association study (GWAS) is another strategy to identify genes associated with resistance to BPH in natural population of rice (Zhou et al., 2021). This method take advantage of ancient recombination events to identify genetic loci underlying complex traits at a relatively high resolution (Zhu et al., 2008). Previously, we detected numerous loci associated with antibiosis to BPH from 1,520 rice varieties (Zhou et al., 2021). The antibiosis levels were evaluated by measuring the bodyweight of insects on rice plants. Since the antibiosis level can not fully reflect the resistance of rice. It is essential to utilize an comprehensive method to evaluate the resistance to BPH and identify more resistance loci.

Genomic selection (GS), also known as genomic prediction (GP), uses genome-wide molecular markers to train models for populations with known phenotypes and genotypes. The trained model predicts the phenotype of individuals possessing only known genotypes, subsequently selecting the most performing individual as a parent for the next generation (Crossa et al., 2017). Compared to molecular marker assisted selection (MAS), which use a set of selected markers to track target genes, GS incorporates molecular markers throughout the genome to predict genomic estimated breeding values (GEBVs) to avoid measurement bias and information loss (Spindel et al., 2015). This strategy effectively tracks and selects minor gene effects while maintaining focus on major genes. Consequently, GS is an effective method for incorporating both major and minor genes into new varieties. Prediction ability (accuracy) is quantified via the correlation between observed phenotypes and predicted GEBVs. The benefits of GS are directly proportional to the prediction accuracy. When the prediction accuracy is high enough, GS can shorten breeding time by increasing the proportion of outstanding offspring in the breeding population. Factors influencing prediction accuracy include marker number, training population sample size, genetic relationship between training and testing populations, statistical models, trait heritability and genetic structure, population structure, and so on (Desta and Ortiz, 2014; Wang et al., 2018c; Guo et al., 2019; Voss-Fels et al., 2019; Xu et al., 2021). However, there is no report of GP on BPH resistance, let alone study to explore the effects of these factors on rice resistance to BPH.

In this study, the BPH resistance of 502 rice varieties was comprehensively evaluated, including resistance score, weight gain rate and honeydew excretion, and more BPH-resistant rice varieties were identified. We also analyzed the correlation between different resistance evaluation methods. GWAS were carried out to identify significantly associated loci in the 502 rice panel and subspecies. Eight resistance candidate genes predicted to encode RLK, NB-LRR or LRR protein on chromosome 11 were identified. Furthermore, we evaluated the predictive ability of GS for BPH resistance and explored the effects of marker number, training population sample size, genetic relatedness between training and testing populations, and statistical models on the predictive accuracy of BPH resistance. The results of our study provide novel BPH-resistant candidate genes and germplasm resources. Estimating the predictive accuracy of GS for BPH resistance are of great importance for the application of GS in developing durable BPH-resistant rice varieties.

## Materials and methods

### Plant materials and BPH insects

The rice materials used in this study comprised of 502 rice varieties randomly selected from 1520 rice varieties (Zhou et al., 2021); taking into account factors, such as the country of origin, eco-cultural type and varietal grouping. The detailed information of these materials can be found in **Supplementary Table 1**. The majority of them were *indica* (227), followed by *circum*-aus (146) and *japonica* (102). There were only 8 accession of *circum*-basmati, and the remaining ones belonged to the admixture type. BPH biotype I was employed as the insect for assessing resistance among the 502 varieties, which were reared on Taichung Native1(TN1) within a controlled greenhouse environment. The temperature inside the greenhouse was maintained at 26 ± 2°C, with an alternating dark period of 8 hours and a photoperiod of 16 hours.

### BPH resistance scores evaluation

The resistance scores of 502 rice varieties against BPH were determined using a modified bulk seedling test following the method of Pathak et al. (1969). Twenty-five seeds of each variety, including the susceptible control variety TN1, were individually sown separately in a 18-cm row and 2-cm row spacing within a 58×38×9 cm seedbox. At the three-leaf stage, weak seedlings were removed and the remaining seedlings were infested with ten second- to third-instar nymphs per seedling. The damage degree of each seeding was evaluated when more than 90% of the control plants TN1 had died, and assigned a resistance score ranging from 1 to 9 based on criteria described by Huang et al. (2001). Higher resistance score indicate susceptibility and lower score indicate resistance. The average resistance score for approximately twenty seedlings was recorded as the resistance score for each variety.

### Evaluation of weight gain rates

Eight seeds of each variety were sown in a 9-cm-diameter plastic cup. At the fifth-leaf stage, newly emerged female BPH adults were weighed using an electronic balance (Shimadzu; Type : AUW120D) and then placed inside a 2×2 cm parafilm bag that had beed securely attached to the leaf sheath of the rice plant. Only insects with initial weight ranging from 1.8 to 2.7 mg were selected for further experimentation. After feeding on the sheath for 48 hours, the insects were removed and reweighted. The weight gain rate was calculated by dividing the insect’s weight gain at 48 hours by its initial body weight. The average weight gain rates of approximately 12 insects was used as the final weight gain rate of a rice variety.

### Honeydew excretion measurements

The honeydew excretion was simultaneously measured with the insect weight. Firstly, the initial weight of the parafilm bag was obtained using the electronic balance. Subsequently, the parafilm bag was securely attached to the tested rice plant and a selected insect was placed inside. After 48 hours, the parafilm bag was reweighed to determine the change in weight, which represented the amount of honeydew excretion. The amount of honeydew excretion of BPHs on each variety was calculated as an average based on approximately twelve insects.

### Statistical analysis

Phenotypic differences among subspecies were performed using the function LSD.test in the R package agricolae (version 1.3-0). The Pearson correlation coefficients (*r*) between resistance levels and antibiosis levels were calculated in stat_cor function in the R package ggpubr. Kurtosis and skewness analysis were conducted using the R package psych.

### Phylogenetic and population structure analysis

The unweighted neighbor-joining tree of 502 rice accessions was constructed based on the identity-by-state (IBS) distance matrix, which was calculated using genome-wide SNPs by PLINK (Chang et al., 2015; version 1.9). and visualized with iTOL software (Letunic and Bork, 2021). The population structure of the 502 varieties was estimated using principal component analysis (PCA) performed by PLINK.

### Genome-wide association study

The SNPs of 502 varieties was filtered by PLINK (Chang et al., 2015) with genotype call rate > 0.8 and minor allele frequency (MAF) > 0.01. A total of 4,452,364 SNPs were used for the subsequent analysis. For the 502 varieties (whole panel), the top seven principal components (PCs) explain 80% of the variance and were used as fixed effects, and the Balding-Nichols kinship matrix (Balding and Nichols, 1995) between each individual was used as random effect for population structure correction. Genome wide association studies was performed using the mixed linear model of software EMMAX (Kang et al., 2010). For *indica* or *japonica*, the top five PCs were used as fixed effects. The Bonferroni correction threshold for multiple tests were used for detecting the genome-wide significant SNPs, which defined as α/N (α = 0.05 and N is the number of SNPs). The *p*-value thresholds for significance were 1.0×10^−8^ for whole panel and subspecies. The manhattan plots and QQ plots were visualized with the R package rMVP (Yin et al., 2021).

### Identification of candidate genes

The linkage disequilibrium (LD) was analysed using PopLDdecay software. The associated locus was defined as a 200 kbp region centered on each associated SNP. Multiple overlapping associated loci were merged into a single locus. The genes within the associated loci were identified through the MSU Rice Genome Annotation Project database, utilizing the Nipponbare genome release 7 (http://rice.plantbiology.msu.edu/). Based on the gene annotation information, candidate genes were determined as those predicted to encode proteins similar to thoes encoded by cloned BPH-resistant genes, including CC-NB-LRR, CC-NB-NB-LRR, CC-NB, LRR and lectin receptor kinase (Shi et al., 2023).

### Selection of marker subsets for genomic prediction

The genotype data utilized the 404K core SNP subset of 3000 Rice Genomes (Wang et al., 2018a). SNPs with a missing rate exceeding 5% and minor allele frequency below 1% were excluded. Following quality control, a total of 268,936 SNPs were obtained. Additionally, the missing SNPs were imputed using Beagle 5.2 with default parameter settings. From the initial set of 268,936 (268K) SNPs set, 12 subsets (0.04K, 0.08K, 0.16K, 0.33K, 0.67K,1.3K, 2.6K, 6.7K, 13K, 26K, 67K, 134K) containing randomly distributed markers were selected utilizing a pseudo-random numbers generator. To minimize the sampling error, the random selections of each subset were repeated 50 times based on the number of SNPs in that particular subset. The numbers of SNPs for the 12 subsets were 42, 84, 168, 336, 672, 1344, 2689, 6723, 13446, 26893, 67234, 134468, respectively.

### Estimation of the heritability

The heritability of the 268K SNPs set was estimated by calculating the ratio of additive genetic variance to total phenotypic variance (Wang et al., 2017). Firstly, the genetic marker was utilized to calculate a genetic relationship matrix (G matrix) using the A.mat function in R package rrBLUP. Subsequently, this G matrix was employed as covariance to estimate the additive genetic variance using the kin.blup function in rrBLUP with the default parameters. The heritability of 268K SNPs was calculated by averaging the results of the 50 random selections. The method for estimating heritability for each marker subset was the same as that of 268K SNPs.

### Genomic prediction models

A total of eight statistical models with different statistical bases were selected to predict the genomic estimated breeding values (GEBVs) for resistance to BPH. Four linear methods, including genomic best linear unbiased prediction (GBLUP), ridge regression best linear unbiased prediction (rrBLUP), bayesian LASSO (BL) and bayesian sparse linear mixed models (BSLMM), were employed. The GBLUP model utilizes the genomic relationship matrix estimated from SNPs and assumes that all SNPs follow a normal distribution. The rrBLUP model is considered equivalent to the GBLUP model (Habier et al., 2007), except that marker scores were inputed into the model. BL and BSLMM represented Bayesian approaches. The marker effects were assumed to follow a double-exponential distribution in BL, and a mixture of two normal distributions in BSLMM. Linear methods may not fully capture non-linear effects such as epistasis and dominance for complex traits (Monir and Zhu, 2018; Azodi et al., 2019). Therefore, three non-linear machine learning methods were also utilized: support vector machine (SVM), random forest (RF), and artificial neural networks (ANN). Additionally, a kinship-adjusted-multiple-loci (KAML) linear mixed model was used, which selects SNPs with big effects as covariates and simultaneously gives larger weights to SNPs with moderate effects and smaller weights to SNPs with little or no effects in the kinship matrix (Yin et al., 2020). These models are the commonly used methods to estimate GEBVs. BSLMM and KAML have been demonstrated to outperform a range of existing methods; thus, we did not exhaustively include other prediction methods into comparison in our study.

### Genomic prediction and cross-validation

The individuals of rice panel was split into a training population that contained 80% of individuals and a validation population that contained the remaining 20%. This produced a training population size of 1179. This procedure was repeated 5 times randomly, and we ensured that the validation populations were the same for all methods. The genomic prediction accuracy was calculated as the average Pearson’s correlation between the GEBVs and observed phenotypes of individuals in validation population. Most statistical models was analysed in R packages. GBLUP and BL were implemented in package BGLR (Pérez and De Los Campos, 2004). 100,000 iterations and 10,000 burn-ins were used to fit the GBLUP and BL model. rrBLUP and BSLMM were implemented in package rrBLUP (Endelman, 2011) and software GEMMA (Zhou and Stephens, 2012), respectively. The three machine learning methods RF, ANN and SVM were analysed in package STGS. KAML were analysed in package KAML (Yin et al., 2020). The default parameters were used for all methods.

### Sampling methods for genetic relatedness analysis

To determine the impact of the genetic relatedness between training and validation population on prediction accuracy, two sampling methods were created for cross-validation based on known population structure, namely stratified sampling and distant sampling. With stratified sampling, all the individuals within each subspecies (*indica*, *japonica*, circum-*aus* and circum-*basmati*) were partitioned into five datasets W1 to W5 with the similar sample sizes. The individuals that fell into W1 were combined across all the subpopulations to build the validation population. In the similar way, each dataset were combined in turn to act as the validation population, and the remaining datasets were combined to act as the training population to estimate prediction accuracy. In stratified sampling, training and validation population contained similar patterns of population stratification, and the genetic relationship between thetraining and validation population was colse. With distant sampling, one subpopulation acted as the validation population and the other subpopulations were combined and served as the training population. These analyses were performed using the 268K SNPs set.

### Selection of training population subsets for genomic prediction

In order to investigate the impact of training population size on prediction accuracy, we select nine subsets from the training population based on proportions of 2%, 5%, 10%, 20%, 30%, 40%, 50%, 60% and 70% of the total training population size. The corresponding individual numbers of the nine training population subsets were 29, 74, 147, 295, 442, 590, 737, 884, and 1032. For each subset, the remaining individuals of 1,520 inbred lines were used as the validation population. Consider the presence of population structure in rice, three training population design methods were also applied in addition to random sampling, including PEVmean and CDmean and stratified sampling (Guo et al., 2019). The selections of subsets by PEVmean and CDmean were implemented in R package STPGA (version 5.2.1) and GenAlgForSubsetSelectionNoTest function. All parameters were set as default. With stratified sampling, the individual number in each subpopulation was determined by the proportion of subpopulation to the whole panel. The sampling process of each sampling method was repeated 50 times. Genomic prediction was conducted using the 268K SNPs set with GBLUP model.

## Results

### Variations in resistance of rice to BPH

**Figure 1:**
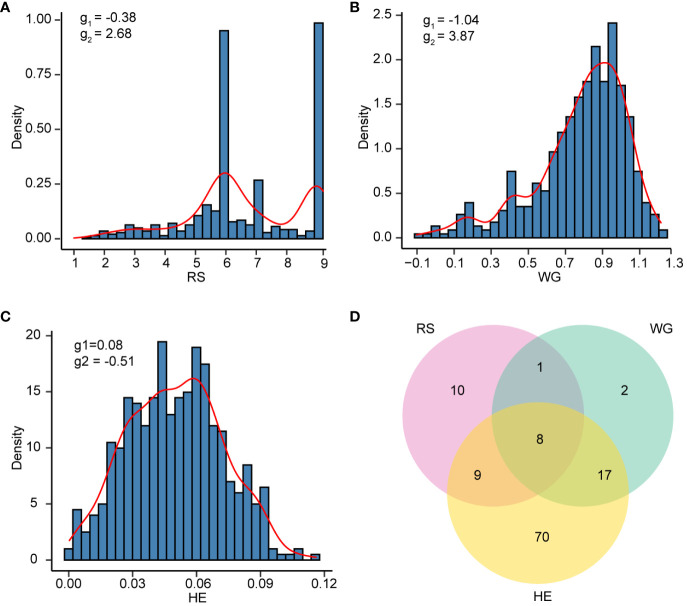
The resistance distribution of 502 rice varieties. **(A-C)** Density histogram of RS **(A)**, WG **(B)**, and HE **(C)**. The skewness (g_1_) and kurtosis (g_2_) are labeled on each plot. **(D)** Venn plot showing the number of varieties with resistant (R) levels in RS, WG and HE.

The results of the phenotypic data analysis for resistance scores (RS), weight gain rates (WG) and honeydew excretions (HE) are presented in **Table 1**. Extensive phenotypic variations were observed among 502 rice varieties in their resistance to BPH. The coefficients of variation (CV) for RS, WG and HE were 0.28, 0.32 and 0.45 respectively, indicating that HE exhibited the highest degree of variation. As indicated by the skewness, RS did not follow a normal distribution but displayed two distinct peaks around values of 6 and 9 on the distribution curve (**Figure 1A**). Among these varieties, 146 (29.08%) varieties showed moderate resistance at RS level 6, while 140 (27.89%) varieties showed extremely susceptible at RS level 9. Both RS and WG demonstrated a right-skewed distribution; however, WG exhibited a greater degree of right skewness compared to RS (**Figures 1A, B**). On the other hand, the distribution of HE was close to a normal distribution (**Figure 1C**).

**Table 1:** Summary statistics of resistance of 502 rice varieties to BPH.

Trait	Subspecies	Number of individuals	Range	Mean ± SD	Skewness	Kurtosis	CV
RS	Whole	502	1.44-9.00	6.63 ± 1.89	-0.38	2.68	0.28
	*indica*	227	1.44-9.00	6.20 ± 1.90	-0.18	-0.31	0.31
	*japonica*	102	3.61-9.00	6.96 ± 1.55	0.27	-1.30	0.22
	circum-*aus*	146	1.70-9.00	6.94 ± 1.98	-0.62	-0.45	0.29
	circum-*basmati*	8	7.00-9.00	8.36 ± 0.92	-0.93	-1.25	0.11
WG	Whole	502	-0.10-1.21	0.78 ± 0.25	-1.04	3.87	0.32
	*indica*	227	0.01-1.21	0.73 ± 0.25	-0.71	-0.02	0.35
	*japonica*	102	-0.10-1.19	0.85 ± 0.20	-1.85	6.04	0.24
	circum-*aus*	146	-0.06-1.19	0.80 ± 0.20	-1.22	1.31	0.33
	circum-*basmati*	8	0.39-1.06	0.86 ± 0.22	-1.48	2.10	0.26
HE	Whole	502	0.0003-0.12	0.05 ± 0.02	0.08	-0.51	0.45
	*indica*	227	0.0003-0.10	0.05 ± 0.02	-0.02	-0.69	0.45
	*japonica*	102	0.0097-0.12	0.06 ± 0.02	0.14	-0.43	0.39
	circum-*aus*	146	0.0014-0.11	0.05 ± 0.02	0.06	-0.61	0.46
	circum-*basmati*	8	0.0181-0.06	0.04 ± 0.02	-0.19	-1.36	0.38

*SD, standard deviation; CV, coefficient of variation.

According to the resistance intervals listed in **Table 2**, the resistance levels of each rice variety were categorized. There were 28 (5.58%), 28 (5.58%) and 104 (20.72%) rice varieties with resistant (R) levels in RS, WG and HE, respectively. Among these traits, eight varieties showed R level to BPH simultaneously. A total of 117 (23.31%) varieties displayed resistance (R level) to BPH (**Figure 1D**).

**Table 2:** Summary information of resistance levels.

Trait	Resistance interval	Resistance level	Number	Percentage of total
RS	(1,3]	R	28	5.58%
	(3,6]	MR	232	46.22%
	(6,9]	S	242	48.21%
WG	(-0.10,0.30]	R	28	5.58%
	(0.30,0.60]	MR	66	13.15%
	(0.60,0.90]	MS	223	44.42%
	(0.90,1.21]	S	185	36.85%
HE	(0,0.03]	R	104	20.72%
	(0.03,0.06]	MR	231	46.02%
	(0.06,0.09]	MS	147	29.28%
	(0.09,0.12]	S	20	3.98%

*R, Resistant; MR, Moderate resistant; MS, Moderate Susceptible; S, Susceptible.

**Figure 2:**
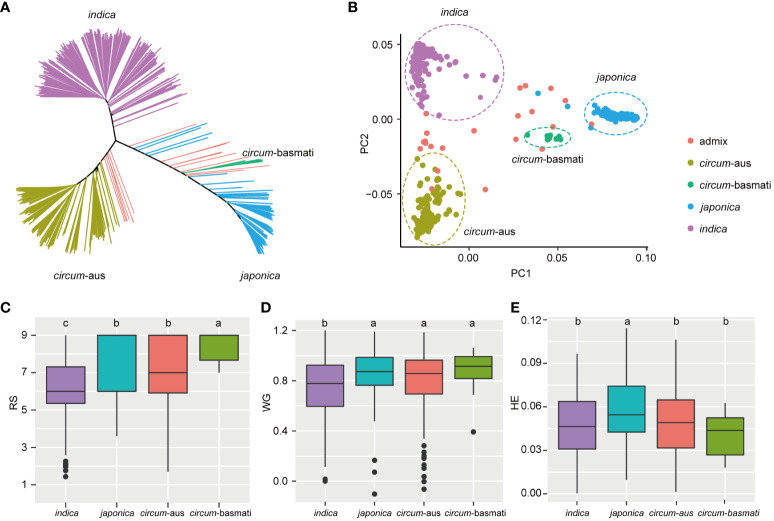
Resistance differences among four subspecies. **(A)** Unweighted neighbor-joining tree based on the IBS distance matrix of 502 rice varieties. Samples are colored according to their subspecies. **(B)** The first two principal components are plotted to display the population structure of 502 rice varieties. *indica*, *japonic*a, circum-*aus* and circum-*basmati* varieties are clustered separately. **(C-E)** Boxplot of RS, WG, and HE of different subspecies. The significant differences between subspecies are indicated by different lowercase letters (p < 0.05, least significant difference test).

Principal component analysis (PCA) revealed the presence of population structure in the 502 rice varieties (**Figures 2A, B**). Subsequently, least significant difference tests were conducted to evaluate the resistance differences among the four subspecies. In terms of RS, *indica* exhibited higher resistance compared to *japonica* and *circum*-aus subspecies, and *circum*-basmati displayed the highest susceptibility (**Figure 2C**). Similarly, for WG, *indica* demonstrated significantly greater resistance than other subspecies (**Figure 2D**). Regarding HE, both *indica* and *circum*-aus along with *circum*-basmati showed significantly resistant than *japonica* (**Figure 2E**). To summarize, rice varieties of *indica* subspecies tend to be more resistant to BPH, which was consistent with previous results (Zhou et al., 2021).

### Correlation analysis between RS, WG and HE

**Figure 3:**
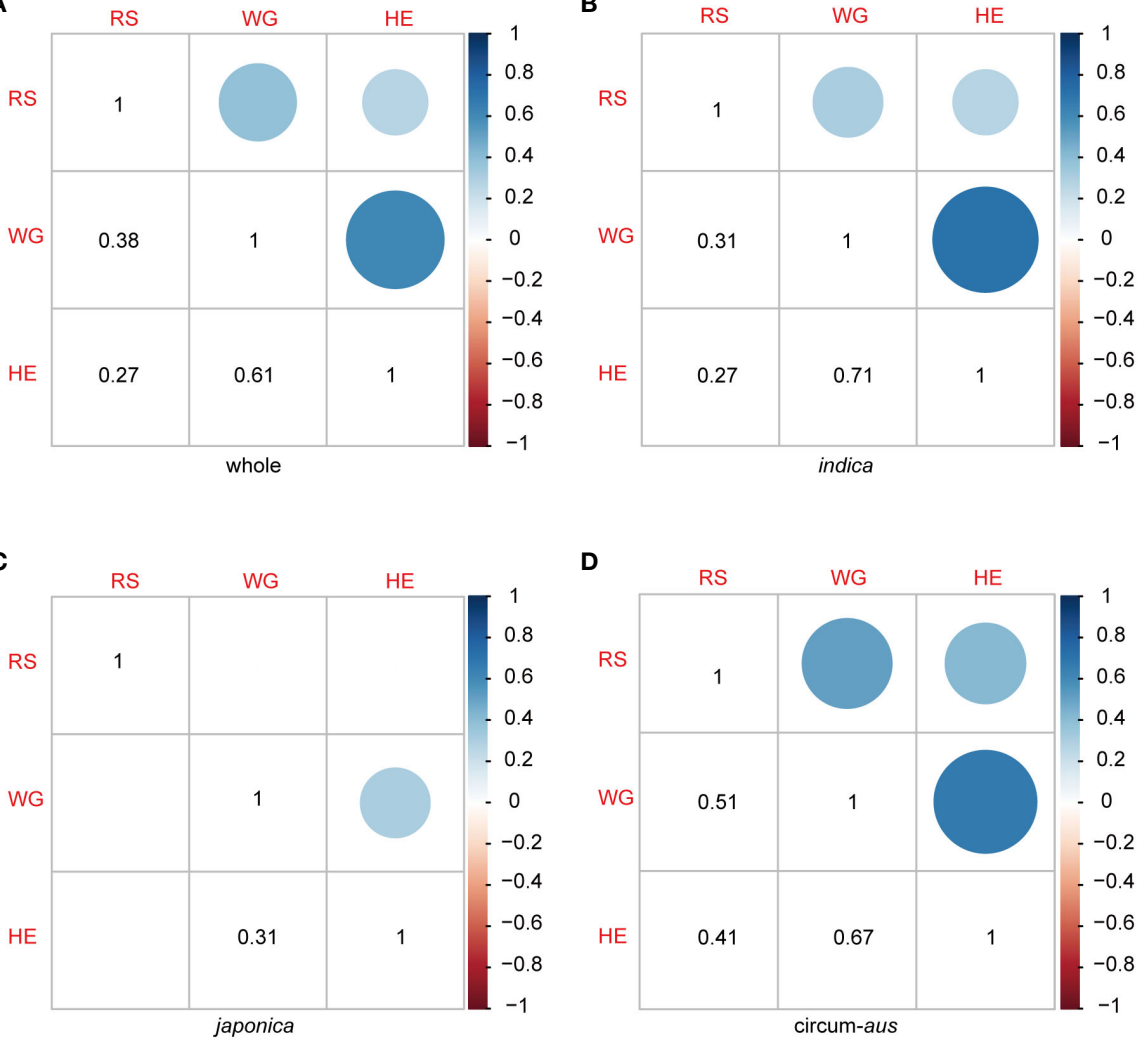
Pearson correlation coefcient matrix among RS, WG and HE in 502 rice varieties **(A)**, *indica* **(B)**, *japonica* **(C)** and circum-*aus* **(D)**.The dots (upper triangle) and numbers (lower triangle) denote the correlation coefcients. Blank squares in the matrix indicate that the correlation between the two corresponding traits are not signifcant (P>0.01).

The results of the correlation analysis between different resistance traits are presented in **Figure 3**. The correlation coefficients between RS, WG, and HE were 0.38, 0.27, and 0.61, respectively (**Figure 3A**). RS of the 502 rice varieties identified in the seedling bulk test exhibited significant correlations with two antibiosis indicators: WG and HE. Specifically, the correlation between RS and WG (*r* = 0.38, *p* < 2.2×10-16) was greater than that between RS and HE (*r* = 0.27, *p* = 5.3×10-10). Furthermore, as two indicators of antibiosis to BPH, the pearson correlation coefficient (*r*) between WG and HE reached 0.61, indicating a strong positive correlation between them (**Figure 3A**).

The correlation patterns varied among the subspecies. There were significant correlations between RS and WG, as well as RS and HE in *indica* and *circum*-aus; however, no significant correlations were observed in *japonica* and *circum*-basmati (**Figures 3B-D**). Moreover, significant correlations were identified between the two indicators of antibiosis in *indica*, *circum*-aus and *japonica* but not in *circum*-basmati (**Figures 3B-D**).

### Associated loci identified by GWAS

**Figure 4:**
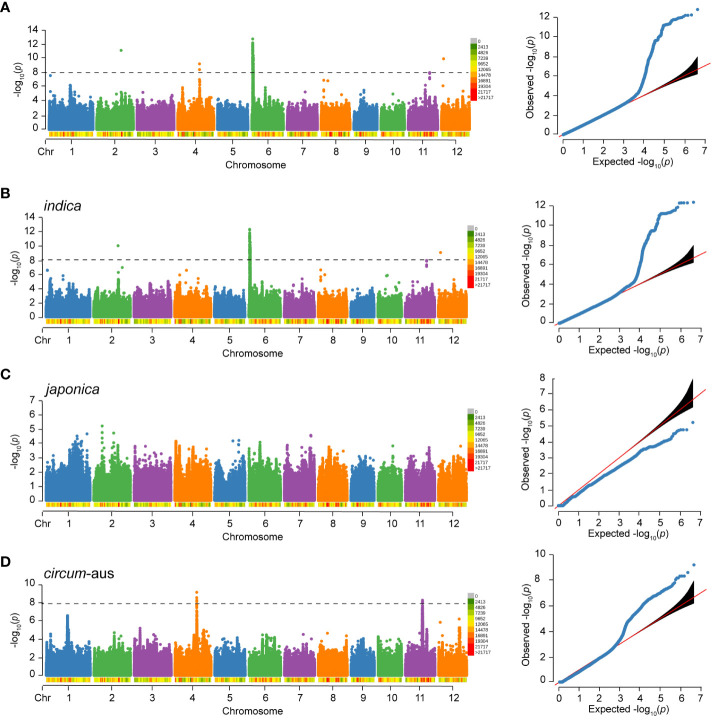
Manhattan plot and Quantile-Quantile (QQ) plot of RS to BPH of 502 rice varieties **(A)**, *indica* **(B)**, *japonica* **(C)** and *circum*-aus **(D)**. The density of SNPs are labeled below the chromosomes. The *p*-value threshold for significance is 1.0×10^-8^.

For RS, a total of 217 significant SNPs (*p*<1.0×10^-8^) were detected on chromosome 2, 4, 6, 11, and 12. Notably, 212 (97.7%) significant SNPs were clustered on chromosome 6 (**Figure 4A**). The genome-wide LD decay rate was estimated at 100 kbp. The associated locus was defined as the 100 kbp region on either side of a significant SNP and multiple overlapping associated loci were merged into a single locus. In total, five loci were associated with RS of 502 varieties. Detailed information regarding these associated loci can be found in **Table 3**.

**Table 3:** Summary information of associated loci obtained by GWAS for RS.

Locus	Population	Chr	Locus region (Mbp)	Number of significant SNPs	Lead SNP	p value	Var(%)	Known R genes	Candidate genes (annotation)
W_1	whole	2	23.86-24.06	1	rs2_23955573	7.26E-12	5.18		
W_2	whole	4	21.27-21.52	2	rs4_21365665	5.78E-10	9.34	*Bph6*	
W_3	whole	6	0.81-1.58	212	rs6_922708	1.78E-13	19.69	*Bph32*/*Bph37*	LOC_Os06g03970 (receptor-like protein kinase)
W_4	whole	11	20.99-21.19	1	rs11_21088754	9.88E-09	4.14		LOC_Os11g35890 (leucine rich repeat protein), LOC_Os11g35960 (leucine rich repeat protein), LOC_Os11g35980 (leucine rich repeat protein), LOC_Os11g36020 (leucine rich repeat protein)
W_5	whoe	12	1.96-2.16	1	rs12_2060801	1.11E-10	10.03		
I_1	*indica*	2	23.86-24.06	1	rs2_23955573	9.86E-11	4.14		
I_2	*indica*	6	0.81-1.58	256	rs6_922708	4.81E-13	13.65	*Bph32*/*Bph37*	LOC_Os06g03970 (receptor-like protein kinase)
I_3	*indica*	12	1.96-2.16	1	rs12_2060801	8.89E-10	6.26		
cA_1	*circum*-aus	4	21.27-21.52	4	rs4_21393633	6.58E-10	5.68	Bph6	
cA_2	*circum*-aus	11	16.64-16.88	11	rs11_16777730	4.95E-09	7.02		LOC_Os11g29030 (NBS-LRR disease resistance protein), LOC_Os11g29050 (NBS-LRR type disease resistance protein), LOC_Os11g29110 (Leucine Rich Repeat protein)

Considering the potential influence of population structure, separate GWAS were performed in subspecies. There were three and two loci associated with RS of *indica* and *circum*-aus, respectively (**Figures 4B, D**, **Table 3**). It was obvious that the associated loci detected in 502 varieties were contributed by *indica* and *circum*-aus. No associated loci were detected in *japonica* (**Figure 4C**). Interestingly, one associated locus on chromosome 11 (cA_2) was specific in *circum*-aus but not in 502 varieties. Overall, six unique loci were significantly associated with RS, and two loci (W_4 and cA_2) were novel loci that had not been previously identified (**Table 3**).

**Figure 5:**
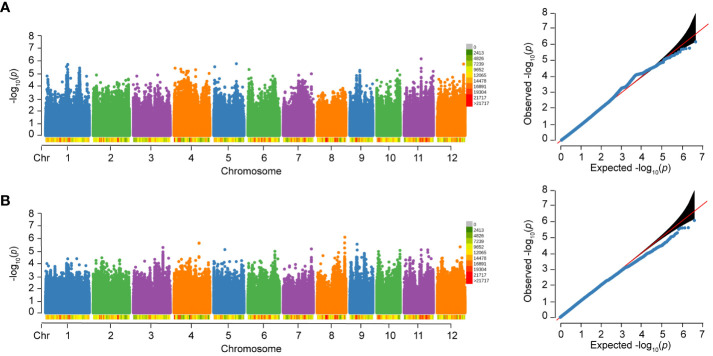
Manhattan plots and Quantile-Quantile (QQ) plots of WG and HE of 502 rice varieties to BPH. **(A)** WG. **(B)** HE.

No significant SNP was detected to be associated with WG and HE in 502 varieties, as shown in **Figure 5**. However, in our previous study with an expanded sample size of 1,520 individuals, we identified 17 loci that were significantly associated with WG. Among the six loci associated with RS, five loci (83.33%) were also associated with WG, and one locus (W_4) is only associated with RS. In addition, separate GWAS were performed in subspecies, and no significant SNP was detected to be associated with WG and HE.

### Identification of resistance candidate genes

**Figure 6:**
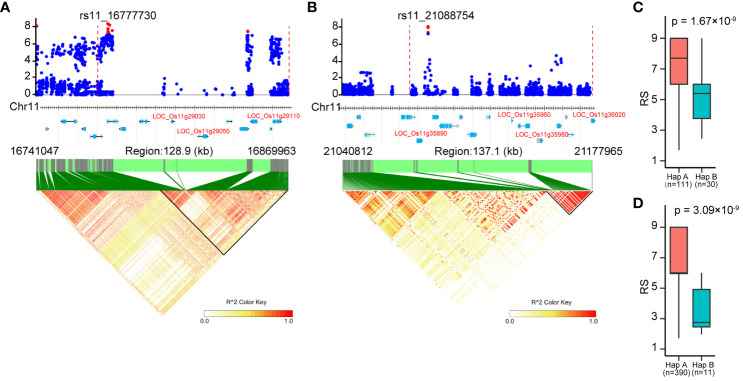
Identification of candidate genes for the associated loci on chromosome 11. **(A)**, **(B)** Local manhattan plots (top) and LD heatmaps (bottom) surrounding the peak SNPs of associated loci cA_2 **(A)** and W_4 **(B)**. The vertical dashed lines in the local manhattan plots indicate the LD blocks. Red dots indicate significantly associated SNPs. All genes in the associated loci are marked at the bottom of the manhattan plots, and the candidate genes are represented in red letters. **(C, D)** The resistance of the two haplotypes based on peak SNP of cA_2 **(C)** and W_4 **(D)**. Significant differences between haplotypes were analyzed by student’s t-test.

There were a total of 331 genes annotated in the associated loci according to the Nipponbare reference genome (**Supplementary Table S2**). These included the known BPH-resistant genes *Bph6*, *Bph32* and *Bph37* on chromosome 4 and 6, confirming the effectiveness of GWAS in identifying BPH-resistant gene. In addition, candidate genes were identified based on their protein domain similarity to the cloned BPH-resistant gene (Shi et al., 2023), resulting in the identification of eight candidate genes (**Table 3**). On chromosome 11, a significant cluster of SNPs was observed in the region spanning from 16.64 to 16.88 Mbp (**Figure 4D**). The local manhattan plot and LD heatmap surrounding the peak SNPs showed that three candidate genes (LOC_Os11g29030, LOC_Os11g29050, LOC_Os11g29110) were localized within a single LD block of approximately 100-kbp size (**Figure 6A**). Another associated locus on chromosome 11 was located in 20.99-21.19 Mbp, with four candidate genes (LOC_Os11g35890, LOC_Os11g35960, LOC_Os11g35980 and LOC_Os11g36020) localized within a 100-kbp LD block spanning from 21.08 to 21.19 Mbp (**Figure 6B**). Notably, there was a significant difference in resistance scores between the two haplotypes based on peak SNP (**Figures 6C, D**). The presence of clustered candidate genes encoding RLK, NBS-LRR or LRR protein on chromosome 11 suggests their potential involvement in BPH resistance.

### Effect of marker number and statistical model on GP accuracy

To validate the usefulness of associations identifed by GWAS in molecular improvement programmes, we performed genomic prediction (GP) and evaluated its predictive ability for BPH resistance. Considering that a larger sample size of 1,520 allowed for the detection of more association loci, we used WG data of 1,520 rice varieties for GP.

**Figure 7:**
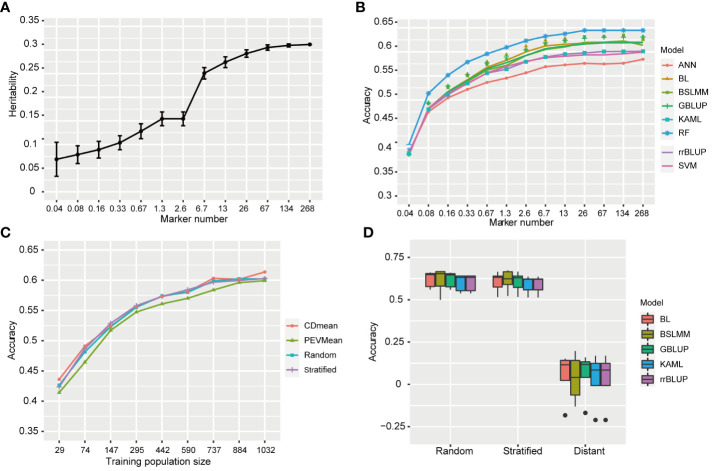
Genomic prediction accuracy for resistance to BPH. **(A)** The heritability of resistance to BPH estimated using 12 SNPs subsets. The standard deviation of 50 repetitions is marked with error bar. **(B)** The prediction accuracy for resistance to BPH by eight statistical models under 268K SNPs set and 12 subsets with different SNP numbers. rrBLUP, ridge regression best linear unbiased predictor; GBLUP, genomic best linear unbiased prediction; BL, bayesian LASSO; BSLMM, bayesian sparse linear mixed models; RF, random forest; ANN, artificial neural network; SVM, support vector machine; KAML, kinship-adjusted-multiple-loci linear mixed model. **(C)** The prediction accuracy for resistance to BPH using different training population sizes by four sampling methods. The training sets were selected by CDmean, PEVmean, stratified sampling and random sampling. Nine different training population sizes (29, 74, 147, 295, 442, 590, 737, 884, and 1032) were used. **(D)** The prediction accuracy for resistance to BPH using random, stratified and distant sampling by five statistical models.

To investigate the effect of marker number on prediction accuracy and determine the minimum number of markers required for predicting resistance to BPH, we selected 12 subsets with randomly distributed markers from the full set of 268,936 (268K) SNPs. This process was repeated 50 times for each subset. The estimated heritability based on the full set (268K) and subsets ranged from 0.069 to 0.312 (**Figure 7A**). The heritability value increased as the number of markers increased. Specifically, there was a rapid increase in estimated heritability when the marker number increased from 2.6 K to 6.7 K, and then it tended to stabilize when the marker number increased to 67 K. The average prediction accuracies using eight statistical models under full set and twelve subsets are shown in **Figure 7B**. The average prediction accuracy ranged from 0.385 to 0.633 and increased as the marker number increased from 0.04K to 26K, subsequently showing minimal improvement. However, there were no significant difference in the prediction accuracy between 6.7K SNPs and 268K SNPs (p<0.05, t-test).

To investigate the effect of statistical model on prediction accuracy, we used eight statistical models with different statistical bases. Among these models, RF achieved an average prediction accuracy of 0.633 when the marker number was 26K, while ANN had the lowest prediction accuracy at 0.576. GBLUP, BSLMM and BL showed similar prediction accuracies, as did KAML, rrBLUP and SVM, which were in the middle level between RF and ANN in terms of prediction accuracy. The average prediction accuracies of RF were significant higher than that of other models (p<0.05, t-test). Therefore, RF outperformed the other models in predicting rice resistance to BPH.

### Effect of training population sizes on GP accuracy

In the studies of phenotypic prediction, the high cost of collecting phenotypic data limits the size of training population. To investigate the influence of training population size on accuracy in predicting resistance to BPH, we successively reduced the size of our training population and evaluated its predictive performance. The prediction accuracies ranged from 0.414 to 0.614 using different training population sizes by four sampling methods, all lower than that achieved by utilizing the entire training population (**Figure 7C**). Generally, an increase in training population size led to improved accuracy. However, when reducing the sample size to 737, there was no significant difference in prediction accuracy compared to utilizing a larger training population consisting of 1179 varieties (p<0.05, t-test).

Among the four different methods employed for selecting the training populations, namely CDmean, PEVmean, stratified sampling and random sampling, comparable levels of prediction accuracies were observed in CDmean, stratified sampling and random sampling. Conversely, PEVmean consistently exhibited lower accuracies.

### Effect of genetic relatedness on GP accuracy

The accuracy of genomic prediction can be influenced by the genetic relatedness between the training and validation population (Wang et al., 2017). To assess its effect on the prediction accuracy of resistance to BPH, we designed stratified sampling and distant sampling based on the known population structure. The prediction accuracies of stratified sampling were significant higher than those achieved with distant sampling (p<0.05, t-test). Specifically, GBLUP estimated an average accuracy of 0.605 for stratified sampling, while distant sampling yielded an average accuracy estimate of only 0.056 (**Figure 7D**). Additionally, We compared the prediction accuracies between stratified and random sampling methods and found no significant differences, suggesting that both training and validation population contained similar patterns of population stratification in random sampling.

## Discussion

BPH is the most destructive insect pest that threatens rice production globally (Dyck and Thomas, 1979). BPH biotype II and III with strengthened virulence emerged with the spread of *Bph1* and *bph2*, respectively (Claridge and Den Hollander, 1980; Kobayashi, 2016). In this study, we evaluated the resistance of 502 rice varieties by evaluating the resistance scores (RS), weight gain rates (WG) and honeydew excretions (HE). A wide range of resistance was observed in the 502 rice varieties. A total of 117 (23.31%) of the 502 rice varieties displayed resistance to BPH. The resistance score from the seedling bulk test was a comprehensive indicator of antibiosis, tolerance and antixenosis (Qiu et al., 2011). Our results showed that RS exhibited significant correlations with the two antibiosis indicators:WG and HE. However, ten rice varieties showed resistant (R) level in RS but moderately susceptible (MS) or susceptible (S) level in WG and HE, suggesting their tolerance or antixenosis to BPH. Generally, tolerance has no selection pressure on BPH biotype (Panda and Heinrichs, 1983). These ten varieties are of potential importance to exploit tolerance genes for controlling BPH and slowing down the emergence of new BPH biotypes. Furthermore, there were thirty-six and three rice varieties in the R level in HE and WG respectively, while RS exhibited S level, indicating that RS is not simply a combination of antibiosis, tolerance, and antixenosis.

The seedling bulk test has the advantage of large-scale and rapid identification of resistance. Combined with its ability to comprehensively assess the level of antibiosis, tolerance and antixenosis, it is often employed for evaluating resistance to BPH and maping resistance gene (Qiu et al., 2011). However, it is difficult to differentiate between antibiosis, tolerance, or antixenosis in a seedling bulk test. In addition, in seedling bulk test, rice varieties to be tested are planted in the box with the susceptible control variety and infested with second- to third-instar nymphs. It’s challenging to maintain the same insect numbers across varieties, and damage scores detection by human vision is less precise than the insect weight in an antibiosis experiment. In the antibiosis experiments, the insect weight was strictly controlled with a balance to ensure the accuracy of the antibiosis. However, tolerance and antixenosis cannot be simultaneously assessed in antibiosis experiment. Therefore, it is necessary to use multiple methods to comprehensively evaluate rice resistance to find more resistant resources.

GWAS were performed on the panel of 502 rice varieties and its subspecies, and 6 loci were associated with RS. However, no loci were significantly associated with WG and HE in these 502 rice varieties. Notably, when the sample size was expanded to include 1,520 rice varieties in our previous study (Zhou et al., 2021), we detected 17 loci assciated with WG. The limited sample size likely contributed to the lower statistical power for detecting loci associated with WG and HE. Among the six loci associated with RS, five (83.33%) were also found to be associated with WG of 1,520 rice varieties, and one locus was newly discovered. These findings suggest that increasing sample size can enhance detection power for identifying BPH-resistant loci; however, it is worth noting that compared to WG and HE traits, RS exhibits higher detection power when a small sample size population was used.

The two associated loci (cA_2 and W_4) on chromosome 11 harbored three and four candidate genes predicted to encode NBS-LRR or LRR proteins. These loci were not mapped before and were completely new loci. It is hypothesized that the two loci may confer tolerance or antixenosis towards BPH infestation based on the fact that they were associated with RS but not WG. The candidate genes were located in close proximity, making it challenging to determine which genes were responsible for BPH resistance. Alternatively, it is possible that these candidate genes function collectively similar to *Bph3*, a cluster of three genes (OsLecRK1-OsLecRK3) to confer resistance (Liu et al., 2015). In conclusion, the identification of these candidate genes within the newly discovered loci provides valuable clues for validating their roles in BPH resistance and facilitating rice breeding for BPH resistance.

We assessed the predictive accuracy of genome selection for BPH resistance using natural populations of 1,520 rice varieties, with the highest predictive accuracy reaching 0.633. The highest prediction accuracy value ranged between 0.31 for rice yield prediction and 0.80 for heading date and plant height prediction, similar to the prediction accuracy of rice flowering time (Onogi et al., 2015; Spindel et al., 2015). Typically, predictive accuracy increases with an increasing number of markers until reaching a platform (Xu et al., 2021). When the SNPs number increased to 26K, the prediction accuracy for BPH resistance remained stable, and there was no significant difference in the prediction accuracy between 6.7K SNPs and 268K SNPs. Therefore, when predicting BPH resistance using a training population consisting of 1179 rice varieties, a minimum of approximately 6.7K SNPs displayed comparable accuracy. Prediction accuracy varied among eight different statistical models tested in this study. Random Forest (RF) achieved the highest prediction accuracy at 0.633; while GBLUP, BSLMM, and BL showed similar accuracies at around 0.618 with only slight differences Despite RF’s superior prediction performance, its computational speed was slower compared to others such as GBLUP and BSLMM.

In genome prediction studies, the high costs of phenotyping restrict the size of training population. As we progressively decreased the size of training population, we observed a corresponding decline in its prediction accuracy, indicating that increasing the size could enhance BPH resistance enhance. However, there was negligible difference in prediction accuracy between 737 and 1179 individuals. Therefore, a training population consisting of 737 individuals is sufficient for predicting resistance to BPH. The accuracy of genome prediction is affected by the genetic relatedness between the training and the validation population (Wang et al., 2017). When the genetic proximity is substantial, the prediction accuracy for BPH resistance is considerably higher compared to cases where it is distant. Hence, optimizing the composition of the training population plays a crucial role in achieving superior prediction accuracy even when there is population stratification. Consequently, for high prediction accuracy, it is important to have a broader genetic diversity within the training population while maintaining close genetic relatedness with the validation population. Additionally, to accurately predict resistance to BPH, one should consider increasing SNP numbers beyond 26K, expanding the training population size beyond 737 individuals, and employing RF models. These findings hold great significance in guiding applications of genome selection towards developing durable BPH-resistant rice.

## Data availability statement

The datasets presented in this study can be found in online repositories. The names of the repository/repositories and accession number(s) can be found in the article/**Supplementary Material**.

## Author contributions

CZ: Writing – original draft, Conceptualization, Data curation, Investigation, Formal analysis, Funding acquisition, Visualization. WJ: Writing – original draft, Investigation. JG: Writing – review & editing. LZ: Writing – original draft, Resources. LL: Writing – original draft, Funding acquisition. SL: Writing – review & editing, Funding acquisition. RC: Writing – review & editing, Resources. BD: Writing – review & editing, Conceptualization, Funding acquisition. JH: Writing – review & editing, Data curation, Formal analysis, Investigation.
